# K_v_7 channel opener retigabine reduces self‐administration of cocaine but not sucrose in rats

**DOI:** 10.1111/adb.13428

**Published:** 2024-08-01

**Authors:** Esteban S. Urena, Cody C. Diezel, Mauricio Serna, Grace Hala'ufia, Lisa Majuta, Kara R. Barber, Todd W. Vanderah, Arthur C. Riegel

**Affiliations:** ^1^ Department of Pharmacology, College of Medicine University of Arizona Tucson Arizona USA; ^2^ Neuroscience Graduate Interdisciplinary Program University of Arizona Tucson Arizona USA; ^3^ Comprehensive Pain and Addiction‐Center (CPA‐C) University of Arizona Health Sciences Tucson Arizona USA; ^4^ The Center of Excellence in Addiction Studies (CEAS) University of Arizona Tucson Arizona USA; ^5^ Department of Neuroscience, College of Science University of Arizona Tucson Arizona USA; ^6^ James C. Wyant College of Optical Sciences University of Arizona Tucson Arizona USA

**Keywords:** cocaine self‐administration, Kv7 channel, retigabine

## Abstract

The increasing rates of drug misuse highlight the urgency of identifying improved therapeutics for treatment. Most drug‐seeking behaviours that can be modelled in rodents utilize the repeated intravenous self‐administration (SA) of drugs. Recent studies examining the mesolimbic pathway suggest that K_v_7/KCNQ channels may contribute to the transition from recreational to chronic drug use. However, to date, all such studies used noncontingent, experimenter‐delivered drug model systems, and the extent to which this effect generalizes to rats trained to self‐administer drugs is not known. Here, we tested the ability of retigabine (ezogabine), a K_v_7 channel opener, to regulate instrumental behaviour in male Sprague Dawley rats. We first validated the ability of retigabine to target experimenter‐delivered cocaine in a conditioned place preference (CPP) assay and found that retigabine reduced the acquisition of place preference. Next, we trained rats for cocaine‐SA under a fixed‐ratio or progressive‐ratio reinforcement schedule and found that retigabine pretreatment attenuated the SA of low to moderate doses of cocaine. This was not observed in parallel experiments, with rats self‐administering sucrose, a natural reward. Compared with sucrose‐SA, cocaine‐SA was associated with reductions in the expression of the K_v_7.5 subunit in the nucleus accumbens, without alterations in K_v_7.2 and K_v_7.3. Therefore, these studies reveal a reward‐specific reduction in SA behaviour and support the notion that K_v_7 is a potential therapeutic target for human psychiatric diseases with dysfunctional reward circuitry.

## Graphical abstract

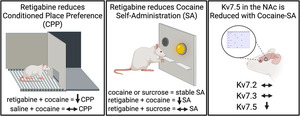

## 1 INTRODUCTION

Repeated exposure to addictive drugs is known to hyperactivate the mesocorticolimbic ventral tegmental area (VTA), nucleus accumbens (NAc) and prefrontal cortex (PFC) brain regions important for the integration of reinforcing stimuli with goal‐directed behaviour.^1^, ^2^ A promising approach to counteract this integration is stabilization of K_v_7/KCNQ voltage‐gated potassium channels, which express different heteromeric combinations of K_v_7.2/K_v_7.3 and K_v_7.3/K_v_7.5 subunits that strongly influence channel properties.^3^, ^4^, ^5^, ^6^, ^7^, ^8^ In neurons, the opening of K_v_7 channels triggers a repolarizing M‐current associated with spike frequency adaptation.^9^, ^10^ Retigabine, a K_v_7 channel opener, stabilizes M‐currents and by positive modulation of the membrane potential restricts the excitability of cortical and mesencephalic dopamine (DA) neurons.^9^, ^10^ As such, targeting K_v_7 channels with retigabine may prove useful in modulating dysfunctional circuitry associated with reward‐related neuropsychiatric and other neurological diseases.^11^, ^12^, ^13^

Furthermore, in vitro and in vivo studies show that retigabine can reduce the release of DA from the terminals of mesencephalic dopaminergic neurons^14^ and reduce the locomotor activity induced by psychostimulants.^15^, ^16^ These actions of retigabine are reversed by the K_v_7 channel blocker XE‐991.^15^, ^17^ However, administration of retigabine alone does not alter exploratory motility and rotarod behaviour,^18^, ^19^ and doses at which retigabine induces anxiolysis can be dissociated from effects on sedation or memory impairment.^18^ To date, most studies using retigabine to modulate drug‐induced changes in DA neurons have used noncontingent methods.^16^, ^20^ Our previous work used an operant model to show that during re‐exposure to drug‐predictive cues, retigabine reduced cocaine‐seeking behaviour and the related changes in pyramidal cell firing and K_v_7 channel currents within the prelimbic PFC.^9^ Existing studies also associate the Kv7.2, Kv7.3, Kv7.4 and Kv7.5 isoforms with the rodent basal ganglia and VTA.^21^, ^22^, ^23^, ^24^, ^25^, ^26^, ^27^ However, it remains unknown whether retigabine alters ongoing drug self‐administration (SA) behaviour, from which these relapse‐like neuroadaptations later emerge.

In this study, we propose that K_v_7 channels act as inhibitory regulators of motivated drug behaviour, thus decreasing the reinforcing actions of cocaine during the maintenance of chronic voluntary SA. We first tested this hypothesis in male Sprague Dawley rats to identify a retigabine dose sufficient to reduce the conditioned preference for cocaine and then determined whether lower, similar or higher doses of retigabine reversibly reduced the operant responding for various doses of cocaine. We showed that retigabine reversibly weakens the motivation for cocaine‐SA at low to moderate doses on a fixed‐ratio or progressive‐ratio (PR) reinforcement schedule. We examined the expression of various K_v_7 channel subunits in relevant mesolimbic regions and provided evidence that relative to sucrose‐SA, chronic cocaine‐SA was associated with a notable reduction in the expression of the K_v_7.5 channel subunit in the accumbens. This study shows that the motivation to self‐administer cocaine is reduced by a potent activator of K_v_7 channels and that the ability to ameliorate this drug behaviour does not necessarily generalize to other nondrug (natural) rewards.

## 2 METHODS

### 2.1 Subjects

Male Sprague Dawley rats (*n* = 120; Envigo) weighing 250–275 g were housed individually in a reverse 12‐h light/dark cycle (light off at 06:00 AM). All procedures performed were preapproved by the Institutional Animal Care and Use Committee of the University of Arizona and according to the Animal Care Guidelines of the National Institutes of Health for the Care and Use of Laboratory Animals. See Data S1.

### 2.2 Drugs

Retigabine (Axon Medchem) was dissolved in saline with 10% Tween‐80 (Sigma‐Aldrich) at concentrations of 2, 5 or 7 mg/mL. Retigabine or vehicle pretreatments were administered 15 min before CPP or SA.^19^ Doses of retigabine were selected on the basis of previous studies showing similar and higher doses produced no significant decrements in a rotarod test and zero maze exploration.^18^, ^19^ As the retigabine solution has a purple colour that is clearly discernable from the vehicle solution, we considered the experimenters to be unblinded. Cocaine hydrochloride (Drug Supply Program of the National Institute on Drug Abuse) was prepared in sterile saline.

### 2.3 Conditioned place preference (CPP)

**FIGURE 1:**
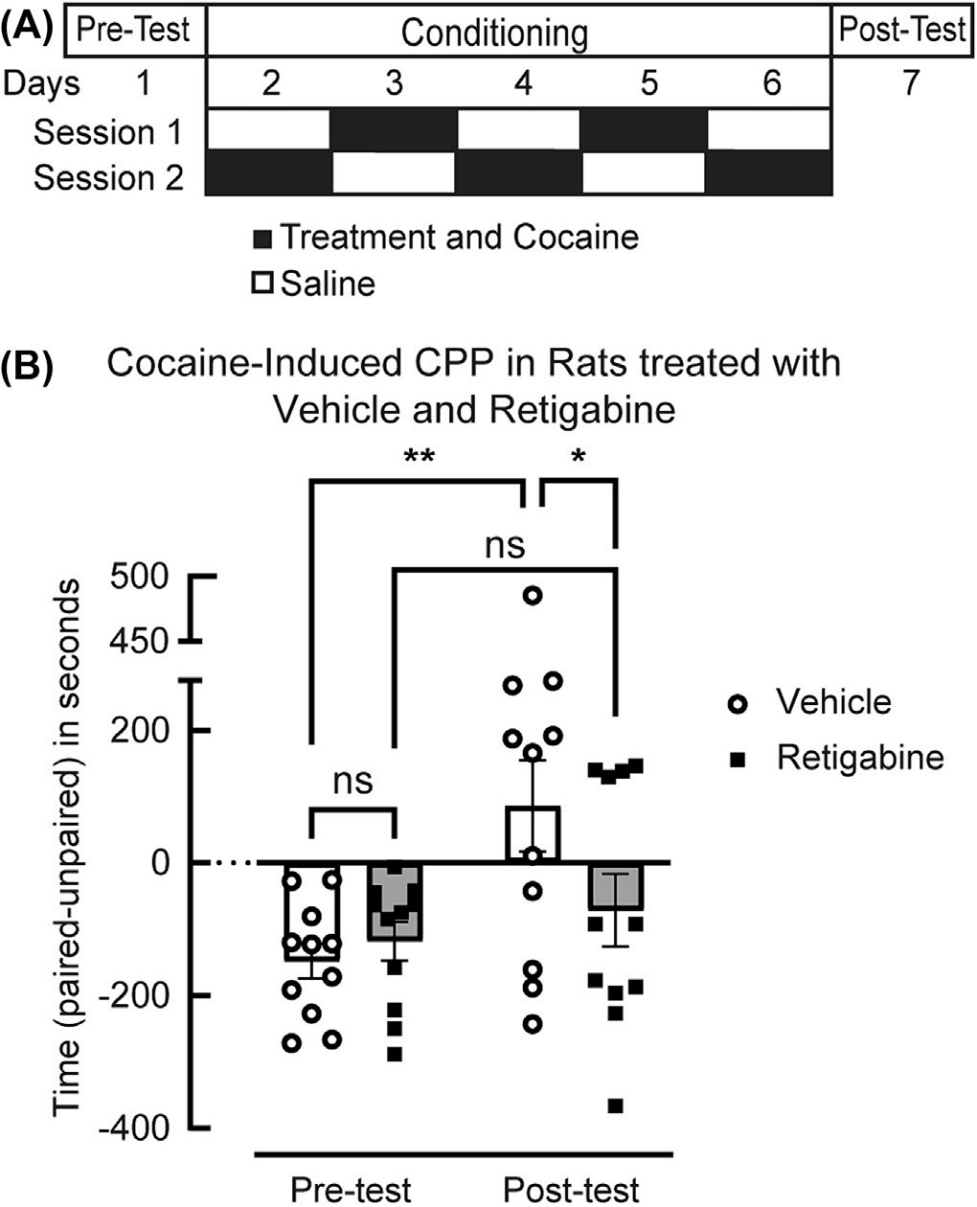
Retigabine reduces the acquisition of cocaine conditioned place preference (CPP). (A) CPP testing included a pretest and 5 days of twice daily intraperitoneal injections of cocaine (black box; 10 mg/kg) and saline (white box; 1 mL/kg) in alternating counterbalanced fashion. At 15 min before cocaine, rats received intraperitoneal pretreatments of either vehicle (1 mL/kg; *n* = 11) or retigabine (5 mg/kg; *n* = 11). On Day 7, rats were tested for side preference. (B) CPP score was calculated as the difference in the time spent in individual chambers during pre‐test and post‐test. A 2W ANOVA indicated an interaction of conditioning with cocaine × retigabine (F_(1, 40)_ = 4.551, *p* = 0.0391). Rats pretreated with vehicle showed a preference for the cocaine‐paired chamber (*t*_(40)_ = 3.530; **p = 0.0011) compared with pre‐test. Rats pretreated with retigabine showed reduced preference for the cocaine‐paired chamber compared with vehicle‐treated controls (*t*_(40)_ = 2.567 *p = 0.0141). For these and all other figures, the error bars indicate the mean ± SEM.

Using our established procedures,^28^ we performed cocaine CPP in a three‐chambered system (San Diego Instruments). Using a biased design, rats were assigned to the least preferred chamber for pairing with cocaine (10 mg/kg, i.p.). Rats were assigned randomly to receive either retigabine or vehicle. Conditioning sessions (30 min) occurred twice a day, with one chamber in the morning (Session 1) and the opposite chamber in the afternoon (Session 2). Daily saline and cocaine sessions were counterbalanced with respect to session (Figure 1A). Pretreatments with retigabine (5 mg/kg, i.p.) or vehicle (10% tween‐80 in saline) occurred 15 min before each cocaine conditioning session. After the five conditioning days, the rats underwent a 15‐min post‐test. CPP score was calculated as the difference in the time spent in individual chambers during pre‐test and post‐test.^29^ Locomotor activity during the pre‐test and post‐test was not evaluated.

### 2.4 Intravenous catheter surgery

Using our published procedures,^9^ rats anaesthetised with isoflurane gas (5% for induction; 2.1%–2.5% for maintenance) received a 22 gauge silastic catheter placed in the right jugular vein. See Data S1.

### 2.5 Cocaine‐SA

Food‐restricted rats underwent cocaine‐SA (0.5 mg/kg/infusion) on a fixed‐ratio 1 (FR1) reinforcement schedule (5‐s infusion and 20‐s timeout post infusion) for 2 h per session, 1 session per day for 6 days per week.^9^ See Data S1.

**FIGURE 2:**
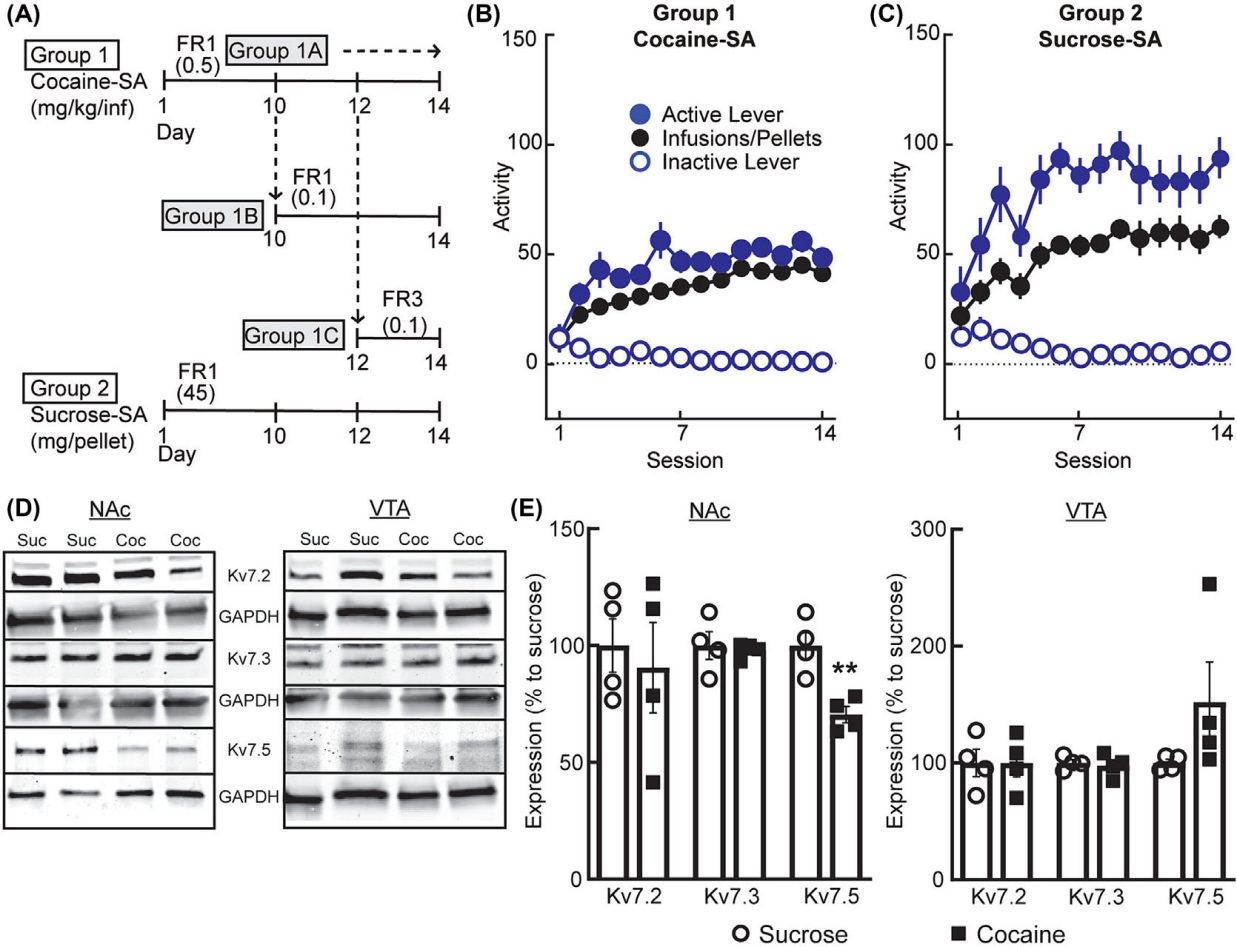
Training for self‐administration (SA) of cocaine or sucrose and expression of the K_v_7 channel prior to retigabine testing. (A) Cohort design for rats learning to self‐administer cocaine (cocaine‐SA, Group 1; 0.5 mg/kg unit dose) or sucrose‐SA (Group 2; 45 mg pellet). After Days 10–12 daily sessions, cocaine‐SA rats remained in the same paradigm (Group 1A, 0.5 mg/kg/infusion; fixed‐ratio 1 [FR1]) or transitioned to a different dose (Group 1B, 0.1 mg/kg/infusion; FR1) or dose and reinforcement schedule (Group 1C, 0.1 mg/kg/infusion; FR3). (B, C) Summary of behavioural responding when rats were presented with levers that resulted in the activation of a light and tone cue paired with (B) a cocaine infusion or (C) a sucrose pellet. (D, E) Western blots of nucleus accumbens (NAc) and ventral tegmental area (VTA) punch lysates from rats that learned to self‐administer cocaine (Group 1, *n* = 4) or sucrose (Group 2, *n* = 4) showing K_v_7 subunit expression (7.2, 7.3, 7.5) normalized to Glyceraldehyde 3‐Phosphate Dehydrogenase (GAPDH) and expressed as a percentage of sucrose controls. (E) In the NAc, Kv7.5 expression decreased, whereas in the VTA, K_v_7 subunit expression did not differ between treatment groups. **p = 0.0052 compared with sucrose‐SA using an unpaired *t*‐test.

**FIGURE 3:**
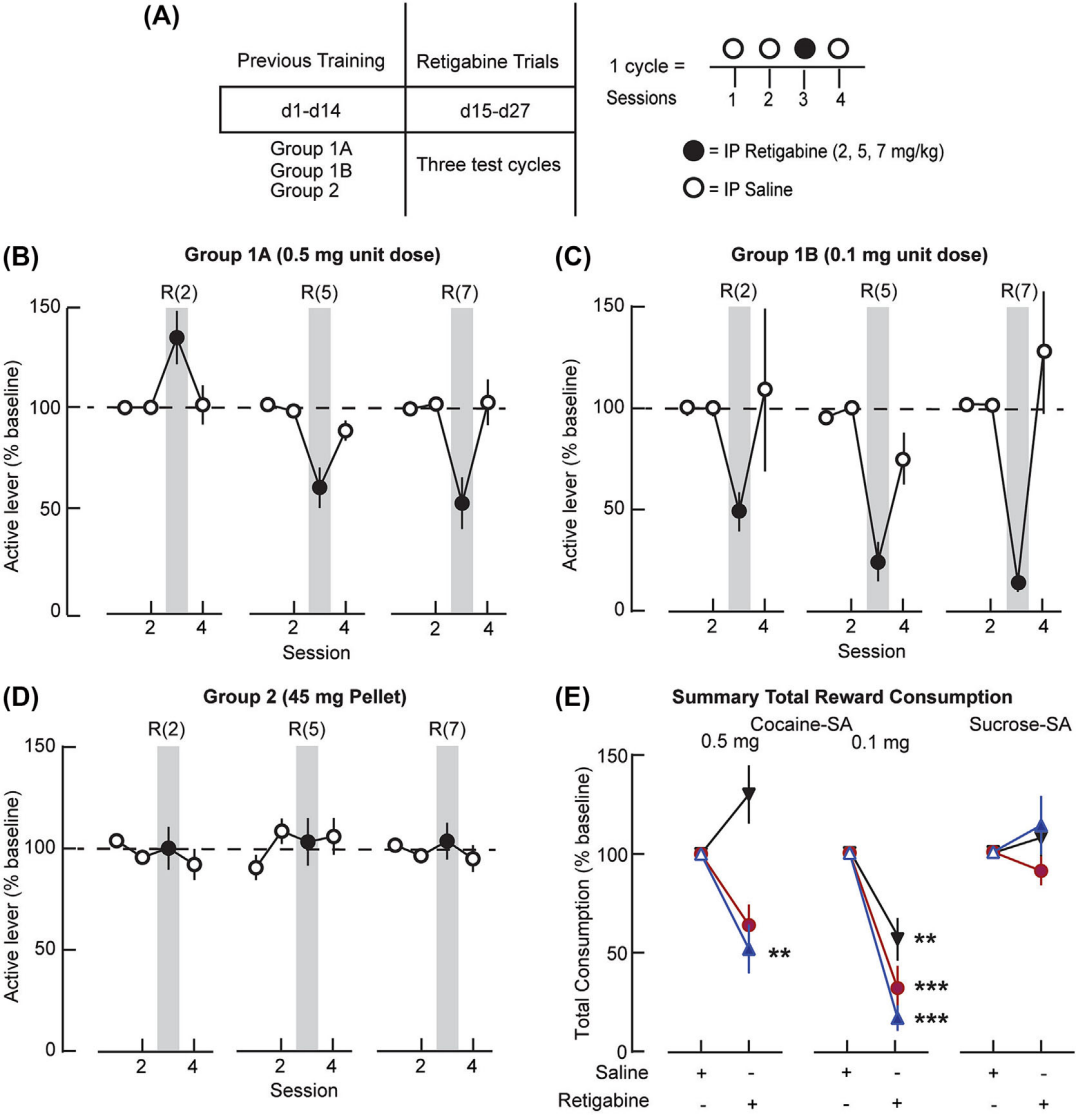
Retigabine reduces cocaine‐ but not sucrose‐self‐administration (SA). (A) Schematic depicting retigabine testing on a fixed‐ratio 1 (FR1). Each group underwent 3 cycles (4 sessions per cycle) of testing with pretreatments with saline (1 mL/kg; Sessions 1, 2 and 4) or increasing doses of retigabine (2, 5, 7 mg/kg, i.p.; Session 3) in a repeated‐measure design administered 15 min before SA. (B, C, D) Summary data showing active lever responding in consecutive sessions (expressed as a percent of baseline) during an FR1 reinforcement schedule for cocaine at unit doses of (B) 0.5 mg (C) 0.1 mg or (D) sucrose (45 mg). Retigabine altered the active‐lever responding for (B, C) cocaine at both unit doses, but not for (D) sucrose. Grey bars indicate retigabine pretreatment (R) at doses denoted by the numbers in parentheses (2, 5, 7 mg/kg, i.p.). (E) Summary of the total reward consumed after pretreatment with saline or different doses of retigabine (▼2, ●5 or 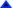
7 mg/kg). Significance compared with saline at the cocaine unit doses of 0.5 mg (7 mg/kg, **p = 0.0133) and 0.1 mg (2 mg/kg, **p = 0.0027; 5 and 7 mg/kg, ****p < 0.0001) using a Šídák's multiple comparison test.

Following the acquisition of cocaine‐SA, animals were divided into three groups (1A, 1B and 1C) (Figure 2A). Before retigabine testing, animals in Groups 1A and 1B continued training on an FR1 (2 h) schedule, at a dose of 0.5 mg/kg/infusion (Group 1A) or a lower dose of 0.1 mg/kg/infusion (Group 1B). Animals were habituated to the (i.p.) pretreatment injections with a minimum of 2 days of saline treatments (1 mL/kg, i.p.) before retigabine and every day thereafter if not receiving retigabine (Figure 3A). The half‐life of retigabine in rodents is about 3 h,^30^ and we allowed a minimum of 2 days between the retigabine tests.

**FIGURE 4:**
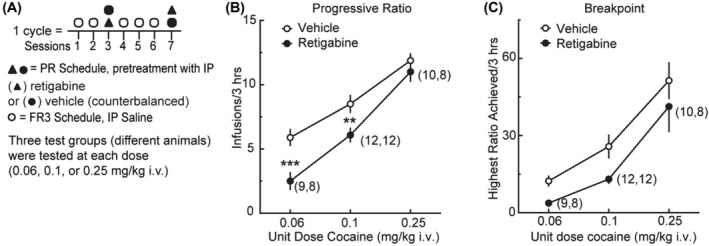
Retigabine reduces the motivation for cocaine in the progressive‐ratio task. (A) Schematic depicting retigabine testing in a progressive‐ratio (PR) schedule of reinforcement. Group 1C rats received pretreatments with saline (1 mL/kg; Sessions 1, 2, 4–6) 15 min prior to session of cocaine‐self‐administration (SA) (0.1 mg unit dose) on the fixed‐ratio 3 (FR3) reinforcement schedule. In Sessions 3 and 7, rats received vehicle (1 mL/kg) or retigabine (5 mg/kg, i.p.) pretreatments 15 min before cocaine (0.06, 0.1 or 0.25 mg/kg) in a PR reinforcement schedule. Separate groups of rats were used for the different doses of cocaine. (B, C) Effects of retigabine on (B) infusions and (C) breakpoints under a PR schedule of cocaine reinforcement at various doses. Numbers in parentheses denote the number of subjects. ***p = 0.0002 (0.06 mg unit dose, infusions), **p = 0.0021 (0.1 mg unit dose, infusions) compared with vehicle using a Šídák's multiple comparison test; in (B), main effect, F_2,29_ = 12.71, *p* < 0.0001; retigabine F_1,24_ = 10.63, *p* < 0.0033; dose × retigabine F_2,24_ = 0.7867, *p* = 0.4667.

In addition to the 12‐session FR1 training (above), animals in Group 1C (Figure 2A) underwent two additional sessions of FR3 (each session 2 h at 0.1 mg/kg/infusion), before transferring to cocaine‐SA on a PR schedule to test with retigabine. On the PR schedule (Figure 4A), three groups of rats self‐administered cocaine at one of three different unit doses (0.06, 0.1 or 0.25 mg/kg/infusion) with 3 days of FR3 between PR sessions (method adapted from Allain et al.^31^). See Data S1.

### 2.6 Sucrose‐SA

Naïve, ad libitum fed rats (*n* = 12) were trained on an FR1 sucrose‐SA paradigm (20 s timeout post infusion) for 3 h per session (Figure 2A), one session per day, 6 days per week as previously described.^9^ See Data S1.

### 2.7 Western blot

Using our published procedures,^9^ we conducted a Western blot analysis to assess the expression of Kv7 channel isoforms in the NAc region (core and shell) and VTA of rats that underwent either cocaine (*n* = 4; Group 1) or sucrose (*n* = 4; Group 2) SA (see Figure 2). Membranes were blocked at room temperature for 1 h in Tris‐buffered saline with 5% Bovine Serum Albumin (BSA) and 0.1% Tween20 then probed overnight with primary antibodies. See Data S1.

### 2.8 Statistical analysis

All values are expressed as means ± SEM. The normality of the data was assessed and found to be normally distributed according to a D'Agostino and Pearson omnibus normality test. Differences between groups were assessed using *t*‐tests, one‐way or two‐way repeated measures (RM) mixed‐effects model. When significant main effects were obtained, appropriate post‐hoc comparisons between groups were made using a Fisher's LSD, Šídák's or Tukey multiple comparisons test. Significance was set at α = 0.05. All analyses were performed on Prism version 9.4.0 (GraphPad, La Jolla, CA).

## 3 RESULTS

### 3.1 Retigabine decreases cocaine CPP

Retigabine reduces DA neuron firing associated with psychostimulants,^20^ but its functional impact on cocaine‐SA remains unknown. To evaluate this, we used a cocaine CPP (Figure 1A) assay to first confirm a retigabine dose that effectively reduced the acquisition of a noncontingent reward behaviour. For 5 days, we pretreated rats with retigabine (5 mg/kg, i.p.) or vehicle 15 min before daily counterbalanced treatments of cocaine or saline. A mixed effects ANOVA showed a significant interaction of conditioning × retigabine (F_(1, 40)_ = 4.551, *p* = 0.0391). During a post‐test, rats pretreated with vehicle showed a preference for the cocaine‐paired chamber (*t*_(40)_ = 3.530; **p = 0.0011) compared with the pre‐test. When animals were pretreated with retigabine, rats spent significantly less time in the cocaine‐paired chamber compared with vehicle‐treated controls following cocaine conditioning (Figure 1; *t*_(40)_ = 2.567; *p = 0.0141). These behavioural results identified a dose of retigabine sufficient to reduce noncontingent reward behaviour.

### 3.2 Training to self‐administer cocaine or sucrose

To determine whether retigabine would also reduce the operant responding for a drug relative to a natural reward, we trained separate groups of male rats (Figure 2A) to respond for cocaine (0.5 mg/kg/infusion; Figure 2B) or sucrose pellets (Figure 2C) on an FR1 schedule of reinforcement for at least 10 consecutive sessions. The rats readily learned to distinguish between active and inactive cocaine levers (lever: F_1,136_ = 243.0, *p* < 0.0001; time: F_5.424,515.3_ = 2.078, *p* = 0.0611; interaction F_13,1235_ = 5.965, *p* < 0.0001) or sucrose (lever: F_1,22_ = 86.72, *p* < 0.0001; time: F_3.095,61.90_ = 2.674, *p* = 0.0533; interaction F_13,260_ = 6.544, *p* < 0.0001), indicating that the processing of reward reinforcement was intact. Because the effectiveness of retigabine may depend on the dose of cocaine or reinforcement schedule, we subdivided rats undergoing cocaine‐SA (Figure 2A) into three treatment groups to acclimate the animals to the different dose (0.5 mg/kg/infusion, FR1: Group 1A, *n* = 29; 0.1 mg/kg/infusion, FR1: Group 1B *n* = 14) and/or reinforcement schedule (0.1 mg/kg/infusion, FR3: Group 1C, *n* = 32). Comparing across cocaine and sucrose groups, we found no differences in the percentage of animals that reached the final criterion (Chi‐square χ^2^_(3)_ = 1.584 [*n* = 98], *p* = 0.6630), indicating that the animals were ready for retigabine testing.

To determine whether the cocaine experiences altered the protein expression of K_v_7 channel subunits relative to sucrose, we sacrificed a subset of Group 1A sucrose‐SA and cocaine‐SA rats at 24 h after the final SA session for a Western blot analysis (Figure 2D). We evaluated the main subunits of the neuronal K_v_7 family in the NAc and VTA tissue homogenates. In the NAc of cocaine‐SA rats, the expression levels of K_v_7.5 were reduced (Figure 2E; t_6_ = 4.277, *p* = 0.0052), whereas the K_v_7.2 (t_6_ = 0.4209, *p* = 0.6885) and K_v_7.3 (t_6_ = 0.3348, *p* = 0.7491) were not. In contrast, in the VTA region, the K_v_7.2 (t_6_ = 0.0074, *p* = 0.9943), the K_v_7.3, t_6_ = 0.4033, *p* = 0.7007) and K_v_7.5 (t_6_ = 0.7127, *p* = 0.5028) were similar between the drug and nondrug reward groups (Figure 2E). Based on these results, we next determined whether the retigabine activation of inhibitory K_v_7 channels would reduce the operant responding for cocaine or sucrose.

### 3.3 Retigabine testing with cocaine‐ or sucrose‐SA

Using a 3‐cycle within‐subject design (with 1 dose of retigabine per cycle; Figure 3A), we compared the effects of saline with 3 doses of retigabine on the maintenance of reward SA behaviour. In Sessions 1 and 2, we injected rats with saline 15 min prior to SA sessions to habituate them to i.p. pretreatments. In Session 3, we pretreated rats with retigabine (2, 5 or 7 mg/kg, i.p.) to test for changes in SA behaviour. In cocaine‐SA groups (Figure 3B,C), the substitution of retigabine for saline reduced active lever‐pressing (Figure 3B 0.5 mg/kg/infusion: treatment F_1,18_ = 8.218, *p* = 0.0103; dose F_1.637,29.47_ = 7.579, *p* = 0.0037; interaction F_1.921,17.29_ = 16.06, *p* = 0.0001; Figure 3C 0.1 mg/kg/infusion: treatment F_1,10_ = 55.79, *p* = 0.0001; dose F_1.756,17.56_ = 1.190, *p* = 0.3218; interaction F_1.917,9.585_ = 2.299, *p* = 0.1539). Retigabine at 7 mg/kg, ip decreased cocaine responses by twofold at 0.5 mg/kg/infusion (Figure 3B) and sixfold at 0.1 mg/kg/infusion (Figure 3C). In contrast, in sucrose rats (Figure 3D) active lever‐pressing was nearly unchanged in response to the same doses of retigabine (treatment F_1,11_ = 0.2843, *p* = 0.6045; dose F_1.795,19.75_ = 1.560, *p* = 0.2350; interaction F_1.771,19.48_ = 3.045, *p* = 0.0759). We did not observe differences in inactive lever responding for any of the groups (Figure S2). These results indicated that activation of K_v_7 channels could decrease the ability to maintain a SA pattern for cocaine, without altering similar responses for a natural reward.

To understand if the amount of reward consumed also decreased, we adjusted the change in the active lever responses for the total amount of reinforcer consumed in mg/kg and then expressed the data as a percentage of the baseline response to the preceding saline pretreatment (Figure 3E). A two‐way RM model with factors of pretreatment (saline or retigabine) and dose of retigabine revealed for the highest dose of cocaine (Figure 3E, left) a significant main effect of retigabine treatment (F_1,90_ = 5.795, *p* = 0.0181) and dose (F_2,90_ = 11.40, *p* < 0.0001), with an interaction between treatment and dose (F_2,90_ = 11.40, *p* < 0.0001). The lower dose of cocaine (Figure 3E, middle) showed a similarly significant effect of retigabine (F_1,10_ = 71.71, *p* < 0.0001) and dose (F_2,20_ = 6.295, *p* = 0.0076), with an interaction between treatment and dose (F_2,10_ = 6.295, *p* = 0.0170). However, sucrose animals (Figure 3E, right) showed no such response to retigabine (treatment: F_1,11_ = 0.2371, *p* = 0.6359; dose: F_2,22_ = 1.287, *p* = 0.2962; interaction: F_2,22_ = 1.287, *p* = 0.2962). These results indicated that the retigabine responses differed between the cocaine‐ and sucrose‐SA groups.

With the higher dose of cocaine (Figure 3E, left; Group 1A: 0.5 mg/kg/infusion), responding on the active lever increased after 2 mg/kg retigabine and decreased after retigabine at 5 and 7 mg/kg. Compared with saline pretreatment, only the reduction in cocaine consumption with the 7 mg/kg dose of retigabine differed significantly (Šídák's multiple comparison test, t_180_ = 3.379, *p* = 0.0133). The changes in cocaine consumption with the 2 and 5 mg/kg dose of retigabine did not differ significantly compared with saline (Šídák's multiple comparison test, 2 mg/kg t_180_ = 2.564, *p* = 0.1551; 5 mg/kg t_180_ = 2.815, *p* = 0.0783). On the contrary, with the lower dose of cocaine (Figure 3E, middle; Group 1B: 0.1 mg/kg/infusion), cocaine consumption decreased significantly relative to the saline baseline in all rats (2 mg/kg, t_20_ = 4.589, *p* = 0.0027; 5 mg/kg, t_20_ = 6.541, *p* < 0.0001; 7 mg/kg, t_20_ = 7.789, *p* < 0.0001). The retigabine reduction was ~3.5‐fold greater with 7 mg/kg than 2 mg/kg retigabine (t_30_ = 4.938, *p* = 0.004). Other changes with retigabine did not differ significantly from the saline baseline. These findings indicated dose‐dependent actions of retigabine in the lower (Group 1B: 0.1 mg/kg/infusion) doses of cocaine.

Because the retigabine action did not generalize across reinforcers, we inspected the cumulative records of individual animals within the various reinforcer SA sessions (Figure 3B–D) and made some general observations. First, we noted that the reductions related to retigabine in the distribution of the inter‐event intervals (Figure S1) for cocaine appeared dose‐dependent. That is, in general for the cocaine sessions, the total number of infusions decreased as the dose of retigabine increased. However, retigabine failed to affect the distribution of inter‐event intervals at either concentration of cocaine. Second, we observed that these changes were not evident in sucrose‐SA rats. As an additional step, we evaluated responding on the inactive lever during pretreatment with saline for the three increasing doses of retigabine. A 2W RM ANOVA did not indicate significant differences related to retigabine in inactive lever responses within cocaine (Groups 1A and 1B) or sucrose (Group 2) rats (Figure S2). Taken together, these results indicate that the pharmacological enhancement of K_v_7 channels with retigabine reduced cocaine‐SA, but the extent to which this occurred depended on both the dose of cocaine and retigabine. Lastly, the effects of retigabine did not generalize to sucrose reward.

### 3.4 Retigabine testing in a progressive‐ratio task

The PR schedule provides an index of reinforcement efficacy.^32^ As a final test, we used this approach to determine whether enhancing the activity of K_v_7 channels altered the motivation to earn a cocaine reward. Using Group 1C rats, we compared PR responses (Figure 4A) after a pretreatment with saline (1 mL/kg, i.p.) versus retigabine (5 mg/kg, i.p.) in a repeated measure design. We used separate groups of animals to test the different doses of cocaine (0.06, 0.1 or 0.25 mg/kg/infusion). All rats reached the breakpoint prior to the 3‐h time limit. During PR tests, we found that responding for cocaine was dose‐dependent and that rats responded less for cocaine at lower doses (Figure 4B; 2W RM mixed‐effects model: F_2,29_ = 35.79, *p* < 0.0001). There was also a main effect of treatment (Figure 4A), indicating that when averaged between cocaine doses, rats exhibited lower infusion rates when treated with retigabine than when treated with vehicle (F_1,25_ = 29.01, *p* < 0.0001). The difference between retigabine and vehicle was robust at a low dose of cocaine, but only modest at the highest dose of cocaine tested. There was a significant interaction (Figure 4B) of the dose of cocaine with retigabine (F_2,25_ = 3.627, *p* = 0.0414). A post‐hoc Šídák's multiple comparison tests indicated that retigabine reduced infusions at the two lowest unit doses of cocaine (Figure 4B; 0.06 mg unit dose: t_25_ = 4.696, *p* = 0.0002; 0.1 mg unit dose: t_25_ = 3.857, *p* = 0.0021; 0.25 mg unit dose: t_25_ = 0.9256, *p* = 0.7421). Across the groups, the cocaine breakpoints increased as a function of the cocaine dose (main effect, F_2,29_ = 12.71, *p* < 0.0001; Figure 4C). However, the dose–response curve for cocaine was significantly decreased in response to retigabine (retigabine F_1,24_ = 10.63, *p* < 0.0033; dose × retigabine F_2,24_ = 0.7867, *p* = 0.4667; Figure 4C). There were no main effects for the responses of the inactive lever. In summary, retigabine significantly reduced cocaine intake over time and reduced incentive motivation for the drug. In contrast, at the doses tested, retigabine produced little measurable change in sucrose‐SA.

## 4 DISCUSSION

We identified K_v_7 channels as a regulator for the targeted control of chronic cocaine‐SA. On a fixed‐ratio reinforcement schedule, systemic treatments of retigabine dose‐dependently reduced lever‐pressing for cocaine but not the ‘natural’ reward, sucrose. These observations led us to pharmacologically evaluate K_v_7 channel activation during a PR schedule of reinforcement, determining how retigabine shaped motivational responses at different doses of cocaine. Indeed, pretreatments with retigabine obstructed the motivational effects of cocaine at low to moderate doses. Therefore, we posit that, in combination with counselling and behavioural therapies, targeting K_v_7 channels may be a useful strategy for reducing the early stages of drug use and complementing other treatments aimed at recovery during prolonged withdrawal.^33^ The present findings extend our previous research demonstrating that direct activation of cortical K_v_7 channels with retigabine reduced relapse‐like behaviours^9^ and align with recent accumbal work examining experimenter‐delivered drug behaviours.^11^

### 4.1 Retigabine reduces cocaine CPP

To confirm an in vivo dose that would be effective in operant experiments, our research demonstrated a systemic dose of retigabine that blocked the acquisition of cocaine CPP. The reduction resembled that previously reported with flupirtine,^34^ a less selective structural precursor of retigabine that activates K_v_7.2/7.3 and other channels.^35^ The behavioural reduction with retigabine may reflect decreased DA transmission, as similar doses of retigabine reportedly decreased the enhancement of striatal and cortical DA levels associated with noncontingent administration of cocaine,^16^ which appear necessary for the acquisition of CPP.^36^

### 4.2 Retigabine actions in cocaine‐ or sucrose‐SA behaviour

We found that at lower doses of cocaine, K_v_7 channel activation reduced operant responses that had been learned and repeatedly reinforced in an FR paradigm. Although previous studies have not investigated this using operant behaviour, retigabine reduced ethanol consumption in a two‐bottle choice study^37^ and alcohol phenotypes associated with K_v_7 channels.^38^ Although the underlying mechanisms of these and our studies remain to be determined, the critical role of phasic mesocortical DA signalling in reward‐conditioning, learning, and SA behaviour has been intensively investigated for many years.^39^, ^40^, ^41^, ^42^ DA depletion or receptor antagonism consistently impairs performance in instrumental tasks.^43^, ^44^, ^45^

In addition to the examination of fixed‐ratio responses, we confirmed the action of retigabine by measuring the motivational changes to obtain cocaine in a PR reinforcement schedule.^46^ After confirming a previous PR study showing that 0.06–0.25 unit doses supported cocaine‐SA,^31^ we showed that retigabine was more effective at lowering PR responses in lower doses of cocaine. Earlier and more recent perspectives on PR encompassing the perception of opportunity costs and decision making^44^, ^47^ agree with work showing that the breakpoint is elevated when DA transmission is enhanced and attenuated when DA transmission is inhibited or depleted.^43^, ^45^, ^48^, ^49^, ^50^ PR responses for cocaine follow the same pattern.^32^, ^51^

Unlike cocaine, retigabine did not reduce sucrose‐SA on a fixed‐ratio schedule of reinforcement, which was relevant given that both rewards are associated with cue‐induced seeking^52^ and DA neuron activation.^53^ However, although DA release is time‐locked to approach behaviour for both cocaine and sucrose, DA levels quickly normalize during SA of sucrose, but not cocaine.^54^, ^55^ Furthermore, unlike sucrose‐SA, rats with a history of chronic cocaine‐SA do not show the normal decrease in the firing of VTA DA neurons that occurs with learning.^56^ Furthermore, while cocaine‐SA produces persistent long‐term potentiation (LTP) in VTA DA neurons, sucrose‐SA does not.^57^ Although retigabine‐LTP studies have not been reported in VTA DA neurons, in vivo administration of retigabine or flupirtine prevents hippocampal LTP and this effect was blocked by the selective K_v_7 channel antagonist XE991.^58^ Furthermore, our previous work and others showed that direct cortical application of retigabine blocked the seeking for cocaine but not sucrose, the former of which requires VTA DA signalling in the prelimbic cortex.^59^, ^60^

### 4.3 Expression of K_v_7 channel subtype after rewards

Our results confirmed earlier studies showing the expression of K_v_7 channel subunits in NAc and VTA.^27^, ^61^, ^62^ Unlike other reports linking decreases in brain K_v_7.2/K_v_7.3 protein levels with alcohol withdrawal^63^ or a depressive phenotype,^13^, ^24^, ^27^ we found no measurable differences between the drug and non‐drug reward groups in the expression of K_v_7.2/K_v_7.3. Our observed reduction in NAc K_v_7.5 expression was interesting given that chronic SA of psychostimulants and opiates upregulate cAMP‐PKA signalling in NAc,^64^, ^65^ and in culture systems, PKA phosphorylation robustly enhances K_v_7.5 containing channels.^66^, ^67^, ^68^ Chronic upregulation of cAMP‐PKA signalling is associated with reward tolerance and dependence that drive SA.^65^ However, over time, chronic overactivation of cAMP‐PKA signalling could produce homeostatic decreases in K_v_7.5 protein expression. How this would impact overall mesolimbic signalling is unclear, but in the hippocampus, reduced expression of inhibitory channels containing K_v_7.5 is associated with increased excitability.^69^, ^70^, ^71^

## 5 CONCLUSIONS

Our behavioural data align with published behavioural and electrophysiology work causally linking K_v_7 channel‐mediated inhibition to reductions in DA neuron activity.^10^ Doses of retigabine similar to those used in our study reduced the frequency of VTA action potential firing and reduced psychostimulant‐induced increases in DA release from the terminals.^15^, ^20^ In hippocampal and VTA DA cells, retigabine more potently suppressed burst firing activity than basal neuron firing.^20^, ^72^ Therefore, a reasonable scenario is that retigabine activation of K_v_7 channels attenuated excessive dopaminergic neurotransmission in the mesolimbic system, to reduce cocaine‐SA.

Although our data provide a more inclusive evaluation of retigabine and SA is a well‐validated model with predictive value for medications that effectively treat cocaine dependence, the utility of retigabine for human substance abuse disorder is unclear. Whereas preclinical studies often use acute medication pretreatments to evaluate SA, most human studies instead assess cocaine craving or ‘high’.^73^ In controlled clinical trials, cocaine‐SA appears difficult to diminish and may differ at different stages of the addiction cycle. Encouraging examples that decrease cocaine use include modafinil, baclofen and buprenorphine, although the latter two are limited by the issues of dose or enhancement of cocaine intoxication.^73^ Although retigabine was voluntarily withdrawn from the market in 2017 due to limited use and side effects, including skin discoloration, its success in recent clinical trials suggests potential utility for lateral sclerosis and depression.^74^, ^75^, ^76^, ^77^ Nevertheless, retigabine also affects Kv2 channels and GABA_A_ receptors.^78^, ^79^, ^80^ More selective versions of this drug may be promising for the treatment of hyperexcitability diseases.^81^ Taken together, our work emphasizes the potential value of future clinical trials with retigabine and further identification of its mechanisms relevant to drug SA.

## AUTHOR CONTRIBUTIONS

**Esteban S. Urena**: Conceptualization; methodology; data collection; writing—original draft; writing—review and editing. **Cody C. Diezel**: Conceptualization; methodology; data collection; writing—review and editing. **Mauricio Serna**: Data curation; data collection; writing—original draft; writing—review and editing. **Grace Hala'ufla**: Data collection. **Kara R. Barber**: Data collection; data curation; conceptualization; methodology; writing—original draft. **Lisa Majuta**: Data collection; methodology; supervision; writing—review and editing. **Todd W. Vanderah**: Writing—reviewing and editing. **Arthur C. Riegel**: Conceptualization; writing—review and editing; supervision.

## CONFLICT OF INTEREST STATEMENT

The authors declare that they have no known competing financial interests or personal relationships that could have appeared to influence the work reported in this paper.

## Supporting information


**Figure S1.** Changes in the inter‐event interval during self‐administration of various reinforcers following pretreatment with saline (1 ml/kg, i.p.) and increasing doses of retigabine (2, 5, 7 mg/kg i.p.). Self‐administration occurred on a FR1 schedule of reinforcement for cocaine at unit doses of **(*A*)** 0.5 mg and **(*B*)** 0.1 mg or **(*C*)** sucrose (45 mg). Note the decline in cocaine **(*A* and *B*)** compared to sucrose **(*C*)**, with increasing doses of retigabine. All pretreatments occurred 15 minutes prior to behavioral testing. The analysis was conducted as in (PMID: 10755745), and rats lacking active lever responses were excluded from Inter‐Event Interval analysis. The numbers in paratheses (*n/n*) represent the number of rats included in the Inter‐Event Interval analysis from the total number of animals in the experiments. See main text for details on experimental designs (Group 1A, 1B and Group 2).

**Figure S2.** Evaluation of within session lever pressing activity for reinforcers following pretreatment with saline (1 ml/kg, i.p.) and increasing doses of retigabine (2, 5, 7 mg/kg, i.p.). The reinforcers included **(A)** cocaine at 0.5 mg unit dose, **(B)** cocaine at 0.1 mg unit dose, and **(C)** sucrose (45 mg). Response measures included **(1)** active lever presses, **(2)** inactive lever presses, and **(3)** the latency for the active lever responding, defined as the amount of time elapsed between the start of the SA session and the first press of the active lever. Within the sessions for the two different unit doses of cocaine, active lever responding decreased following retigabine pretreatments. **(A)** At a cocaine unit dose of 0.5 mg, responding on the **(A1)** active lever showed a main effect of retigabine dose (F_(3,62= 2.922)_ p = 0.0408) and time (F_(4.014,248.9_) = 2.525, p=0.0413), with a significant interaction (F_(69,1426)_= 1.401, p=0.0185). In contrast, responding on the **(A2)** inactive lever showed no discernible differences (dose F _(3,62)_= 0.8013, p=0.4979; time F_(1.428,88.55)_= 2.601, p=0.0968; dose x time F _(69,1426)_= 0.9258, p=0.6495). **(A3)** Latency measurements varied significantly (ANOVA F _(3,57)_= 4.258, p=0.0088) and pretreatments of retigabine at 7mg/kg (i.p.) produced greater latency than pretreatment with saline (*p = 0.0453) or retigabine at 2 mg/kg (**p=0.0068). For cocaine at a unit dose of **(B)** 0.1 mg, responding on the **(B1)** active lever also showed main effect of retigabine dose (F_(3,35= 3.312)_, p=0.0311) and time (F_(6.239,218.4)_= 3.378, p=0.0029), but no interaction (F_(69,805)_= 0.8216, p=0.8476). Responding on the **(B2)** inactive lever showed no significant differences (dose, F _(3,35)_= 2.345, p=0.0896; time, F _(8.648,301.9)_= 1.198, p=0.2974; dose x time, F_(69,803)_= 1.028, p=0.4182). **(B3)** Latency measurements for cocaine at the 0.1 mg unit dose showed no significant differences (F _(3,32)_= 0.6666, p=0.5788). In contrast to cocaine at the 2 unit doses, responding for sucrose showed no significant effect of retigabine dose on responding on **(C1)** active levers (dose, F _(3,44)_= 1.473, p = 0.2350; time, F _(11.96,526.1)_= 16.88, p<0.0001; dose x time, F _(105,1540)_= 1.938, p<0.0001), **(C2)** inactive levers (dose, F_(3,44= 2.176)_, p=0.1043; time, F_(7.462,328.3)_= 1.113, p=0.3540; dose x time, F _(105,1540)_= 1.078, p=0.2820) or **(C3)** latency (F _(3,44)_= 0.9197, p=0.4392). Pretreatments occurred 15 minutes prior to behavioral testing on a FR1 reinforcement schedule. In A3, B3, C3, rats with no measurable level pressing activity were excluded from latency calculations. The numbers (n/n) in paratheses represent the number of rats included in the analysis from the total number of animals run in the experiments. Statistical comparisons were performed using a 2W RM ANOVA with a Tukey's multiple comparison test or 1W ANOVA with a Tukey's multiple comparison test.

**Data S1.** Supporting Information.

## Data Availability

Data will be uploaded to https://sites.arizona.edu/riegel-lab/data upon publication of the manuscript on request.
