# Supplementary material for: Kv7 channel opener retigabine reduces self‐administration of cocaine but not sucrose in rats
Source: Addict Biol. 2024 Aug 1;29(8):e13428. doi: 10.1111/adb.13428 (PMC11292668; doi:10.1111/adb.13428)
Supplement: Supplementary file 1 — Figure S1. Changes in the inter‐event interval during self‐administration of various reinforcers following pretreatment with saline (1 ml/kg, i.p.) and increasing doses of retigabine (2, 5, 7 mg/kg i.p.). Self‐administration occurred on a FR1 schedule of reinforcement for cocaine at unit doses of (A) 0.5 mg and (B) 0.1 mg or (C) sucrose (45 mg). Note the decline in cocaine (A and B) compared to sucrose (C), with increasing doses of retigabine. All pretreatments occurred 15 minutes prior to behavioral testing. The analysis was conducted as in (PMID: 10755745), and rats lacking active lever responses were excluded from Inter‐Event Interval analysis. The numbers in paratheses (n/n) represent the number of rats included in the Inter‐Event Interval analysis from the total number of animals in the experiments. See main text for details on experimental designs (Group 1A, 1B and Group 2). [file ADB-29-e13428-s003.docx]

**Fig. S1** Changes in the inter-event interval during self-administration of various reinforcers following pretreatment with saline (1 ml/kg, i.p.) and increasing doses of retigabine (2, 5, 7 mg/kg i.p.). Self-administration occurred on a FR1 schedule of reinforcement for cocaine at unit doses of **(*A*)** 0.5 mg and **(*B*)** 0.1 mg or **(*C*)** sucrose (45 mg). Note the decline in cocaine **(*A* and *B*)** compared to sucrose **(*C*)**, with increasing doses of retigabine. All pretreatments occurred 15 minutes prior to behavioral testing. The analysis was conducted as in (PMID: 10755745), and rats lacking active lever responses were excluded from inter-event interval analysis. The numbers in paratheses (*n/n*) represent the number of rats included in the inter-event interval analysis from the total number of animals in the experiments. See main text for details on experimental designs (Group 1A, 1B and Group 2).

**Reference**

Brebner K, Phelan R, Roberts DC. Effect of baclofen on cocaine self-administration in rats reinforced under fixed-ratio 1 and progressive-ratio schedules. *Psychopharmacology (Berl)*. 2000;148(3):314-321. doi:10.1007/s002130050056
