# Supplementary material for: Kv7 channel opener retigabine reduces self‐administration of cocaine but not sucrose in rats
Source: Addict Biol. 2024 Aug 1;29(8):e13428. doi: 10.1111/adb.13428 (PMC11292668; doi:10.1111/adb.13428)
Supplement: Supplementary file 2 — Figure S2. Evaluation of within session lever pressing activity for reinforcers following pretreatment with saline (1 ml/kg, i.p.) and increasing doses of retigabine (2, 5, 7 mg/kg, i.p.). The reinforcers included (A) cocaine at 0.5 mg unit dose, (B) cocaine at 0.1 mg unit dose, and (C) sucrose (45 mg). Response measures included (1) active lever presses, (2) inactive lever presses, and (3) the latency for the active lever responding, defined as the amount of time elapsed between the start of the SA session and the first press of the active lever. Within the sessions for the two different unit doses of cocaine, active lever responding decreased following retigabine pretreatments. (A) At a cocaine unit dose of 0.5 mg, responding on the (A1) active lever showed a main effect of retigabine dose (F(3,62= 2.922) p = 0.0408) and time (F(4.014,248.9) = 2.525, p=0.0413), with a significant interaction (F(69,1426)= 1.401, p=0.0185). In contrast, responding on the (A2) inactive lever showed no discernible differences (dose F (3,62)= 0.8013, p=0.4979; time F(1.428,88.55)= 2.601, p=0.0968; dose x time F (69,1426)= 0.9258, p=0.6495). (A3) Latency measurements varied significantly (ANOVA F (3,57)= 4.258, p=0.0088) and pretreatments of retigabine at 7mg/kg (i.p.) produced greater latency than pretreatment with saline (*p = 0.0453) or retigabine at 2 mg/kg (**p=0.0068). For cocaine at a unit dose of (B) 0.1 mg, responding on the (B1) active lever also showed main effect of retigabine dose (F(3,35= 3.312), p=0.0311) and time (F(6.239,218.4)= 3.378, p=0.0029), but no interaction (F(69,805)= 0.8216, p=0.8476). Responding on the (B2) inactive lever showed no significant differences (dose, F (3,35)= 2.345, p=0.0896; time, F (8.648,301.9)= 1.198, p=0.2974; dose x time, F(69,803)= 1.028, p=0.4182). (B3) Latency measurements for cocaine at the 0.1 mg unit dose showed no significant differences (F (3,32)= 0.6666, p=0.5788). In contrast to cocaine at the 2 unit doses, responding for sucrose [file ADB-29-e13428-s001.docx]

**Fig. S2** Evaluation of within session lever pressing activity for reinforcers following pretreatment with saline (1 ml/kg, i.p.) and increasing doses of retigabine (2, 5, 7 mg/kg, i.p.). The reinforcers included **(A)** cocaine at 0.5 mg unit dose, **(B)** cocaine at 0.1 mg unit dose, and **(C)** sucrose (45 mg). Response measures included **(1)** active lever presses, **(2)** inactive lever presses, and **(3)** the latency for the active lever responding, defined as the amount of time elapsed between the start of the SA session and the first press of the active lever. Within the sessions for the two different unit doses of cocaine, active lever responding decreased following retigabine pretreatments.

**(A)** At a cocaine unit dose of 0.5 mg, responding on the **(A1)** active lever showed a main effect of retigabine dose (F_(3,62= 2.922)_ p=0.0408) and time (F_(4.014,248.9)_ = 2.525, p=0.0413), with a significant interaction (F_(69,1426)_= 1.401, p=0.0185). In contrast, responding on the **(A2)** inactive lever showed no discernible differences (dose F _(3,62)_= 0.8013, p=0.4979; time F_(1.428,88.55)_= 2.601, p=0.0968; dose x time F _(69,1426)_= 0.9258, p=0.6495). **(A3)** Latency measurements varied significantly (ANOVA F _(3,57)_= 4.258, p=0.0088) and pretreatments of retigabine at 7mg/kg (i.p.) produced greater latency than pretreatment with saline (*p=0.0453) or retigabine at 2 mg/kg (**p=0.0068).

For cocaine at a unit dose of **(B)** 0.1 mg, responding on the **(B1)** active lever also showed main effect of retigabine dose (F_(3,35= 3.312)_, p=0.0311) and time (F_(6.239,218.4)_= 3.378, p=0.0029), but no interaction (F_(69,805)_= 0.8216, p=0.8476). Responding on the **(B2)** inactive lever showed no significant differences (dose, F _(3,35)_= 2.345, p=0.0896; time, F _(8.648,301.9)_= 1.198, p=0.2974; dose x time, F_(69,803)_= 1.028, p=0.4182). **(B3)** Latency measurements for cocaine at the 0.1 mg unit dose showed no significant differences (F _(3,32)_= 0.6666, p=0.5788).

In contrast to cocaine at the 2 unit doses, responding for sucrose showed no significant effect of retigabine dose on responding on **(C1)** active levers (dose, F _(3,44)_= 1.473, p=0.2350; time, F _(11.96,526.1)_= 16.88, p<0.0001; dose x time, F _(105,1540)_= 1.938, p<0.0001), **(C2)** inactive levers (dose, F_(3,44= 2.176)_, p=0.1043; time, F_(7.462,328.3)_= 1.113, p=0.3540; dose x time, F _(105,1540)_= 1.078, p=0.2820) or **(C3)** latency (F _(3,44)_= 0.9197, p=0.4392).

Pretreatments occurred 15 minutes prior to behavioral testing on a FR1 reinforcement schedule. In A3, B3, C3, rats with no measurable level pressing activity were excluded from latency calculations. The numbers (n/n) in paratheses represent the number of rats included in the analysis from the total number of animals run in the experiments. Statistical comparisons were performed using a 2W RM ANOVA with a Tukey’s multiple comparison test or 1W ANOVA with a Tukey’s multiple comparison test
