# Supplementary material for: Kv7 channel opener retigabine reduces self‐administration of cocaine but not sucrose in rats
Source: Addict Biol. 2024 Aug 1;29(8):e13428. doi: 10.1111/adb.13428 (PMC11292668; doi:10.1111/adb.13428)
Supplement: Supplementary file 3 — Data S1. Supporting Information. [file ADB-29-e13428-s002.docx]

**SUPPLEMENTAL METHODS**

**Subjects**

Male Sprague Dawley rats (n=120; Envigo) weighing 250-275g were housed individually in a reverse 12-hour light / dark cycle (light off at 06:00 am). Rats were divided into two groups: fed ad libitum and food restricted. Ad libitum rats had non-restricted access to food and water; food-restricted rats had free access to water and were food restricted to maintain 80% of the body weight of ad libitum rats. All procedures performed were preapproved by the Institutional Animal Care and Use Committee of the University of Arizona and according to the Animal Care Guidelines of the National Institutes of Health for the Care and Use of Laboratory Animals.

**Intravenous catheter surgery**

Using our published procedures (PMID: 29636392), rats anesthetized with isoflurane gas (5% for induction; 2.1-2.5% for maintenance) received a 22 gauge silastic catheter placed in the right jugular vein. Silk sutures were used to secure catheters to the vein prior to being exteriorized to the midscapular region of the back and attached to a vascular access button (Instech, VABR1B/22). During surgery rats received an analgesic (Ketorolac 2 mg/kg, i.p.) and antibiotic (Gentamicin 8 mg/kg, i.p.). After surgery, rats received ketorolac (2 mg/kg, i.p.) and recovered 4-7 days with daily catheter flushes of a heparinized solution with gentamicin (8mg/kg) (Henry Schein, part # 1098195). During SA-training, catheters were flushed daily with 0.1 ml of 500u/ml heparin dissolved in sterile saline followed by a 0.1 ml lock of heparin (300u/ml).

**Cocaine-SA**

Food-restricted rats underwent cocaine-SA (0.5 mg/kg/infusion) on a fixed-ratio 1 (FR1) reinforcement schedule (5-s infusion and 20‐s timeout post infusion) for 2h per session, 1 session per day for 6 days per week (PMID: 29636392). Sessions occurred in sound-attenuating operant chambers connected to Med‐PC IV software (Med Associates). During training sessions, a 100µl infusion of cocaine was paired with light and tone cues (white stimulus light above the active lever; 78‐dB, 200‐Hz tone). Lever presses on the 'inactive' lever were counted but lacked programmed consequences. SA criteria was defined as a minimum of 10 infusions within a 25% over/under range for 3 sessions consecutively. Seven rats were excluded from the study due to surgical or behavioral complications.

Following acquisition of cocaine-SA, animals were divided into three groups (1A,1B,1C) (**Fig. 2A**). Before retigabine testing, animals in Group-1A,-1B continued training on a FR1 (2h) schedule, at a dose of 0.5 mg/kg/infusion (Group-1A) or a lower dose of 0.1 mg/kg/infusion (Group-1B). Animals were habituated to the (i.p.) pretreatment injections with a minimum of two days of saline treatments (1 ml/kg, i.p.) before retigabine and every day thereafter if not receiving retigabine (**Fig. 3A**). The half-life of retigabine in rodents is about 3h 30, and we allowed a minimum of 2 days between the retigabine tests.

In addition to the 12-session FR1 training (above), animals in Group-1C (**Fig. 2A**) underwent two additional sessions of FR3 (each session 2h at 0.1 mg/kg/infusion), before transferring to cocaine-SA on a progressive-ratio (PR) schedule to test with retigabine. On the PR schedule (**Fig. 4A**), 3 groups of rats self-administered cocaine at one of 3 different unit doses (0.06, 0.1, or 0.25 mg/kg/infusion) with three days of FR3 between PR sessions (method adapted from PMID: 29085961). The number of active-lever responses required for successive reinforcers increased according to the published formula (PMID: 8451268). The PR sessions ended after reaching a breakpoint or after 3 hours had elapsed, whichever occurred first. Breakpoint was defined as in previous work (PMID: 36386780) as the final completed response ratio before the rat stopped lever pressing enough for an infusion for at least 1 hour.

**Sucrose-SA**

Naïve, ad libitum fed rats (n=12) were trained on a FR1 sucrose-SA paradigm (20s timeout post infusion) for 3 hours per session (**Fig. 2A**), 1 session per day, 6 days per week as previously described ­­9. The operant chambers and procedures were identical to the cocaine-SA described above except that training sessions paired the discrete cues with a sucrose pellet (45 mg, Bio-Serv). After acquisition of sucrose-SA, prior to retigabine, animals were habituated to (i.p.) pretreatment injections with a minimum of two days of saline treatments (1 ml/kg, i.p.) before retigabine and every day thereafter if not receiving retigabine (**Fig. 3A**).

**Western blot**

Using our published procedures (PMID: 29636392), we conducted a Western blot analysis to assess the expression of Kv7 channel isoforms in the nucleus accumbens (NAc) region (core and shell) and ventral tegmental area (VTA) of rats that underwent either cocaine (n=4; Group 1) or sucrose (n=4; Group 2) self-administration (see Figure 2). Using stereotaxic coordinates (Paxinos and Watson, 2014), we visualized and collected 2 mm bilateral punches from 2 mm brain slices and lysed them in a buffer containing 20mM Tris HCl pH 7.5, 150mM NaCl, 2mM EDTA, 0.1% SDS, 1% NP40, 0.25% Deoxycholate, 1mM Sodium Orthovanedate, 1mM PMSF, 1mM NaF, 1X protease inhibitor (Fisher Scientific, #53-514-21SET). Lysates were clarified by centrifugation (15,000 x g, 10 min, 4C), and protein concentration was quantified using the Pierce™ BCA protein assay (Thermo Scientific, catalog # 23225). Samples were prepared for electrophoresis in 2X Laemmli sample buffer (Bio-Rad, catalog # 1610737) and 2-mercaptoethanol according to manufacture specification. Approximately 10 μg of total protein was loaded onto a 4-15% SDS-PAGE gel and transferred to nitrocellulose membranes (0.2 μm). 2 separate gels with sucrose (N=2) and cocaine (N=2) were run in triplicate per region. Membranes were blocked at room temperature for 1 hour in TBST with 5% BSA and probed overnight with primary antibodies against KCNQ2 (1:500, catalog #APC-050, Alomone Labs), KCNQ3 (1:500, catalog #APC-051, Alomone Labs), KCNQ5 (1:500, catalog #APC155, Alomone Labs), and GAPDH (1:1000, catalog #MA1-16757, Invitrogen). The secondary antibodies used were LiCor Goat anti-Rabbit IRDye800 (1:5000) and LiCor Goat anti-Mouse IRDye680 (1:5000). Kv7 channel isoform expressions were normalized to GAPDH, similar to (PMID: 28677679.) Images were captured using an Azure Sapphire imager, and blot densities were analyzed using Azure Spot analysis software.

**References**

PMID: 29636392. Parrilla-Carrero J, Buchta WC, Goswamee P, et al. Restoration of Kv7 channel-mediated inhibition reduces cued-reinstatement of cocaine seeking. *J Neurosci*. 2018;38(17):4212-4229. doi:10.1523/jneurosci.2767-17.2018

PMID: 29085961. Allain F, Bouayad-Gervais K, Samaha AN. High and escalating levels of cocaine intake are dissociable from subsequent incentive motivation for the drug in rats. *Psychopharmacology*. 2018;235(1):317-328. doi:10.1007/s00213-017-4773-8

PMID: 8451268. McGregor A, Lacosta S, Roberts DCS. L-tryptophan decreases the breaking point under a progressive ratio schedule of intravenous cocaine reinforcement in the rat. *Pharmacol Biochem Be*. 1993;44(3):651-655. doi:10.1016/0091-3057(93)90181-r

PMID: 36386780. Scott SN, Ruscitti BA, Garcia R, et al. 5-HT1B receptor agonist enhances breakpoint for cocaine on a progressive ratio (PR) schedule during maintenance of self-administration in female rats but reduces breakpoint for sucrose. *Front Behav Neurosci*. 2022;16:1020146. doi:10.3389/fnbeh.2022.1020146

PMID: 28677679. Sakai A, Saitow F, Maruyama M, et al. MicroRNA cluster miR-17-92 regulates multiple functionally related voltage-gated potassium channels in chronic neuropathic pain. *Nat Commun.* 2017;8:16079. doi:10.1038/ncomms16079
